# Zoledronate rescues immunosuppressed monocytes in sepsis patients

**DOI:** 10.1111/imm.13132

**Published:** 2019-11-21

**Authors:** Loïc Raffray, Ross J. Burton, Sarah E. Baker, Matt P. Morgan, Matthias Eberl

**Affiliations:** ^1^ Division of Infection and Immunity School of Medicine Cardiff University Cardiff UK; ^2^ Department of Internal Medicine Félix Guyon University Hospital of La Réunion Saint Denis France; ^3^ Directorate of Critical Care, Cardiff & Vale University Health Board University Hospital of Wales Cardiff UK; ^4^ Systems Immunity Research Institute Cardiff University Cardiff UK

**Keywords:** gammadelta T‐cells, immunosuppression, monocytes, sepsis, zoledronate

## Abstract

Severe sepsis is often accompanied by a transient immune paralysis, which is associated with enhanced susceptibility to secondary infections and poor clinical outcomes. The functional impairment of antigen‐presenting cells is considered to be a major hallmark of this septic immunosuppression, with reduced HLA‐DR expression on circulating monocytes serving as predictor of mortality. Unconventional lymphocytes like γδ T‐cells have the potential to restore immune defects in a variety of pathologies including cancer, but their use to rescue sepsis‐induced immunosuppression has not been investigated. Our own previous work showed that Vγ9/Vδ2^+^ γδ T‐cells are potent activators of monocytes from healthy volunteers *in vitro*, and in individuals with osteoporosis after first‐time administration of the anti‐bone resorption drug zoledronate *in vivo*. We show here that zoledronate readily induces upregulation of HLA‐DR, CD40 and CD64 on monocytes from both healthy controls and sepsis patients, which could be abrogated by neutralising the pro‐inflammatory cytokines interferon (IFN)‐γ and tumour necrosis factor (TNF)‐α in the cultures. In healthy controls, the upregulation of HLA‐DR on monocytes was proportional to the baseline percentage of Vγ9/Vδ2 T‐cells in the peripheral blood mononuclear cell population. Of note, a proportion of sepsis patients studied here did not show a demonstrable response to zoledronate, predominantly patients with microbiologically confirmed bloodstream infections, compared with sepsis patients with more localised infections marked by negative blood cultures. Taken together, our results suggest that zoledronate can, at least in some individuals, rescue immunosuppressed monocytes during acute sepsis and thus may help improve clinical outcomes during severe infection.

## Graphical abstract

Sepsis is characterised by enhanced susceptibility to secondary infections and poor outcomes as a result of a transient hypoimmune state. Reduced expression of HLA‐DR on circulating monocytes has become a hallmark parameter in those patients that is used both as predictor of mortality and as target for clinical interventions. This study demonstrates that the anti‐bone resorption drug zoledronate has potential in rescuing the immunosuppression in severe sepsis by upregulating the expression of HLA‐DR and other activation markers on monocytes, through a mechanism that depends on the activation of blood γδ T‐cells.

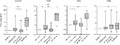

**Abbreviations**
HMB‐PP: (*E*)‐4‐hydroxy‐3‐methyl‐but‐2‐enyl pyrophosphate

## Introduction

The immune system has evolved to sense and fight a myriad of viral, bacterial, fungal and parasitic pathogens. Early inflammatory responses to infection aim to control and clear the causative organism, yet there is emerging evidence in critically ill patients of a subsequent, or parallel, transition to a blunted hypoimmune state.^1^ This acquired immune paresis is believed to render individuals with severe sepsis susceptible to secondary, often hospital‐acquired, infections during recovery from the initial insult.^2^ While previous interventions aimed at targeting the immediate hyperinflammatory phase (‘cytokine storm’) to improve outcomes from sepsis, often with little success, more recent studies focus on utilising immunotherapeutic approaches directed towards this somewhat paradoxical immunosuppression.^2^, ^3^, ^4^ Addressing immunosuppression brings significant survival benefits in cancer,^5^ yet it remains to be shown to be effective in reducing non‐cancer morbidity. The lack of advance in treating infectious diseases is likely due to the challenges in identifying and effectively targeting those patients that would benefit most from intervention, as well as a paucity of safe therapeutic options.

Reduced HLA‐DR expression by monocytes has become a hallmark of sepsis immunosuppression, and is being used not only as clinical predictor of poor outcomes but also as potential target for novel interventions.^6^, ^7^, ^8^, ^9^ A series of clinical trials have aimed at restoring myeloid cell functions, including HLA‐DR levels on monocytes, in sepsis patients by using recombinant cytokines such as interferon (IFN)‐γ and granulocyte macrophage colony‐stimulating factor (GM‐CSF), with mixed results.^10^, ^11^, ^12^ However, despite more than two decades of basic and clinical research in this area, no cytokine therapy has yet been shown to reduce mortality in sepsis patients. Administration of cytokines with pleiotropic roles in the immune system is prone to give rise to unwanted side‐effects. Targeted delivery of immunotherapeutic drugs is therefore desirable to minimise off‐target effects. In this respect, those cellular mechanisms are of particular interest that allow direct and contact‐dependent delivery of effector molecules *in vivo*. One such approach is the delivery of immunomodulatory effector molecules by unconventional T‐cells such as γδ T‐cells.

Vγ9/Vδ2 T‐cells typically comprise 1%–5% of circulating T‐cells in human blood, and are potent producers of pro‐inflammatory cytokines including IFN‐γ, tumour necrosis factor (TNF)‐α and GM‐CSF with activity on myeloid cells.^13^, ^14^, ^15^, ^16^ A major physiological role of Vγ9/Vδ2 T‐cells is the sensing of microbial pathogens that produce the isoprenoid precursor (*E*)‐4‐hydroxy‐3‐methyl‐but‐2‐enyl pyrophosphate (HMB‐PP), which include most Gram‐negative and many Gram‐positive bacteria.^17^ However, Vγ9/Vδ2 T‐cells also readily respond to stimulation with zoledronate, a clinically approved drug used successfully in patients with malignant and non‐malignant bone resorption disorders.^17^, ^18^ This Vγ9/Vδ2 T‐cell response is dependent on monocyte uptake of zoledronate^19^ and involves the interruption of the intracellular mevalonate pathway, leading to accumulation of upstream intermediates such as isopentenyl pyrophosphate and recognition of sensitised monocytes via the butyrophilin‐like molecule BTN3 by the Vγ9/Vδ2 T‐cell receptor.^17^, ^20^, ^21^

In co‐culture experiments, γδ T‐cells activate monocytes, promote their survival, induce upregulation of markers associated with antigen‐presenting cells (APCs) including HLA‐DR, and enhance their APC function.^13^ Clinical evidence in support of such a mechanism comes from our own study in osteoporosis patients demonstrating that administration of zoledronate in otherwise healthy individuals increases expression of APC markers on circulating monocytes *in vivo* and boosts plasma levels of IFN‐γ, TNF‐α and GM‐CSF.^22^ We here tested the potential of zoledronate to activate peripheral monocytes in patients with acute sepsis and provide evidence that zoledronate treatment helps upregulate the expression of HLA‐DR and other APC markers on monocytes *in vitro*. Our findings open potentially new therapeutic avenues toward rescuing the immunosuppressed state of acutely ill patients.

## Materials and methods

### Ethics statement

Recruitment of sepsis patients was approved by the Health and Care Research Wales Research Ethics Committee under reference 17/WA/0253, protocol number SPON1609‐17 and IRAS project ID 231993, and conducted according to the principles expressed in the Declaration of Helsinki. All participants provided written informed consent for the collection of samples and their subsequent analysis. A waiver of consent system was used when patients were unable to provide prospective informed consent due to the nature of their critical illness or therapeutic sedation at the time of recruitment. In all cases, retrospective informed consent was sought as soon as the patient recovered and regained capacity. In cases where a patient passed away before regaining capacity, the initial consultee's approval would stand. Recruitment of healthy adult volunteers was approved by Cardiff University's School of Medicine Research Ethics Committee under reference 18/04.

### Subjects

Sepsis patients were over 18 years old with a diagnosis of sepsis according to the Third International Consensus Definitions for Sepsis and Septic Shock (‘Sepsis‐3’), were cared for in the intensive care unit at the University Hospital of Wales in Cardiff, and were recruited within 36 hr of the presumed onset of the infective illness when they already had or would require arterial cannulation as part of standard treatment. This study cohort comprised a total of *n* = 24 sepsis patients, with an age ranging from 28 to 82 years (median 57 years), 70·8% of which were female. Age‐ and gender‐matched healthy donors served as non‐infected controls (*n* = 19; age range 25–66 years, median 59 years; 57·9% female).

Patients were excluded if they were pregnant or breastfeeding, or were females of childbearing age in whom a pregnancy test had not been performed; if they had severe immune deficiency, for example a diagnosis of AIDS or treatment with anti‐rejection transplant drugs or high‐dose corticosteroids; if they had haematological malignancy or ongoing chemotherapy; if they had severe liver failure (Child's score III or worse); if they were adjudged by the admitting clinician to be unlikely to survive for the duration of the study period regardless of treatment; if they were admitted post‐cardiac arrest; or if they had an underlying impairment of higher function that would make it impossible for informed consent to be given upon recovery (e.g. severe learning disability).

### Cell culture

The culture medium was RPMI‐1640 supplemented with 2 mm l‐glutamine, 1% sodium pyruvate, 50 μg/ml penicillin/streptomycin and 10% foetal calf serum (Invitrogen, Paisley, UK). Peripheral blood mononuclear cells (PBMCs) were isolated from peripheral blood of healthy volunteers using Lymphoprep (Axis‐Shield, Dundee, UK) and cultured with and without 10 µm zoledronate (Zometa; Novartis, Basel, Switzerland) for 16 hr; a combination of 10 ng/ml recombinant IFN‐γ and 20 ng/ml recombinant TNF‐α (both Miltenyi, Woking, UK) was used as positive control. For blocking experiments, anti‐IFN‐γ (B27; Biolegend, London, UK) and sTNFR p75‐IgG1 fusion protein (etanercept/Enbrel; Amgen, Cambridge, UK) were used at 10 μg/ml each. Cell culture supernatants were analysed in duplicate on a CLARIOstar microplate reader (BMG Labtech, Aylesbury, UK), using a sandwich ELISA kit for the detection of TNF‐α (Invitrogen).

### Flow cytometry

Cells were acquired on a BD LSRFortessa flow cytometer (BD Biosciences, Wokingham, UK) and analysed with flowjo version 10 (TreeStar, Ashland, OR, USA). Single cells of interest were gated based on their appearance in side and forward scatter area/height, exclusion of live/dead staining (fixable Aqua; Invitrogen) and surface staining (CD3^−^ CD19^−^ CD14^+^ monocytes, Vδ2^+^ CD3^+^ γδ T‐cells). The following mAbs were used for surface labelling: anti‐CD3 (SP34‐2), anti‐CD64 (10·1) and anti‐TCR‐Vδ2 (B6) from BD Biosciences; anti‐TCR‐pan‐γδ (Immu510) and anti‐CD40 (mAB89) from Beckman Coulter, High Wycombe, UK; and anti‐CD14 (M5E2), anti‐CD86 (IT2·2) and anti‐HLA‐DR (L243) from Biolegend.

### Statistics

Statistical analyses were performed using graphpad Prism 6.0. Variables were tested for normal distribution using D’Agostino−Pearson tests. All statistical tests were two‐tailed; differences were considered significant at *P* < 0·05.

## Results

### Zoledronate treatment of PBMCs induces activation of monocytes

**Figure 1:**
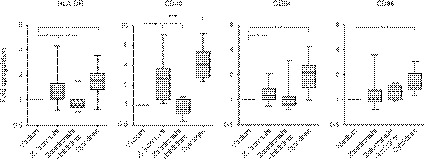
Zoledronate‐induced activation of healthy monocytes. Peripheral blood mononuclear cells (PBMCs) from healthy volunteers were cultured in medium alone or treated overnight with zoledronate in the absence (*n* = 19) or presence of anti‐interferon (IFN)‐γ and sTNFR (Inhibitors; *n* = 15), or with a combination of recombinant IFN‐γ and tumour necrosis factor (TNF)‐α (Cytokines; *n* = 11). Monocyte activation was calculated as fold upregulation of the indicated surface markers (determined as mean fluorescence intensities), in relation to medium controls with a value of 1, and is shown as box and whisker plots (min to max). Data were analysed using Kruskal−Wallis tests, together with Dunn’s multiple comparison *post hoc* tests; asterisks indicate significant differences: *P < 0·05; **P < 0·01; ***P < 0·001.

Our earlier research demonstrated that purified human γδ T‐cells readily induce activation of co‐cultured monocytes, when stimulated with the microbial metabolite HMB‐PP.^13^ We here tested whether this effect on monocytes could be reproduced in PBMC cultures and by replacing HMB‐PP with the clinically approved drug zoledronate. Indeed, overnight stimulation of PBMCs with zoledronate led to a general activation of the monocyte population as evidenced by upregulation of HLA‐DR, CD40 and CD64; upregulation of CD86 failed to reach statistical significance (Fig. 1).

**Figure 2:**
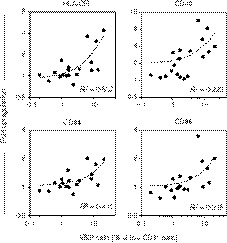
The frequency of blood γδ T‐cells determines the degree of monocyte activation in response to zoledronate. Peripheral blood mononuclear cells (PBMCs) from healthy volunteers (*n* = 19) were cultured in medium alone or treated overnight with zoledronate. Monocyte activation was calculated as fold upregulation of the indicated surface markers (determined as mean fluorescence intensities), in relation to medium controls with a value of 1, and is shown in relation to the frequency of γδ T‐cells in the donors’ blood. Data were analysed using linear regression.

The zoledronate‐dependent effect on monocytes was largely abrogated in the presence of neutralising reagents against IFN‐γ and TNF‐α, indicating that these two cytokines were major drivers of monocyte activation in PBMC cultures treated with zoledronate. As control, incubation of PBMCs with recombinant IFN‐γ and TNF‐α led to a similarly pronounced upregulation of HLA‐DR, CD40, CD64 and CD86 on monocytes as seen in zoledronate‐treated cultures (Fig. 1). In further support of the key role for γδ T‐cells in mediating the observed effects on monocytes, the extent of HLA‐DR upregulation achieved in zoledronate‐treated PBMC cultures correlated directly with the original proportion of Vδ2^+^ T‐cells in the blood from healthy individuals (Fig. 2). Upregulation of CD40, CD64 and CD86 followed a similar trend but was much less pronounced.

### Zoledronate induces activation of monocytes from sepsis patients

**Figure 3:**
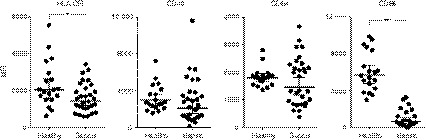
Peripheral monocytes in sepsis patients express reduced levels of HLA‐DR, CD40, CD64 and CD86. Blood from adult sepsis patients (*n* = 30) and from age‐ and gender‐matched healthy volunteers (*n* = 19) was analysed by flow cytometry for expression of the indicated surface markers on peripheral CD14^+^ monocytes. Data are shown as mean fluorescence intensities (MFIs) and were analysed using Mann−Whitney tests; asterisks indicate significant differences: *P < 0·05; **P < 0·01; ***P < 0·001.

**Figure 4:**
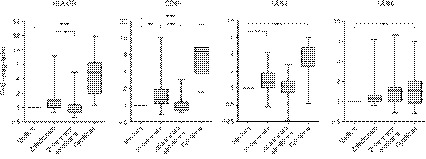
Zoledronate‐induced activation of monocytes from sepsis patients. Peripheral blood mononuclear cells (PBMCs) from patients with acute sepsis were cultured in medium alone or treated overnight with zoledronate in the absence (*n* = 24) or presence of anti‐interferon (IFN)‐γ and sTNFR (Inhibitors; *n* = 24), or with a combination of recombinant IFN‐γ and tumour necrosis factor (TNF)‐α (Cytokines; *n* = 17). Monocyte activation was calculated as fold upregulation of the indicated surface markers (determined as mean fluorescence intensities), in relation to medium controls with a value of 1, and is shown as box plots (min to max). Data were analysed using a Kruskal−Wallis test, together with Dunn’s multiple comparison *post hoc* test; asterisks indicate significant differences: *P < 0·05; **P < 0·01; ***P < 0·001.

**Figure 5:**
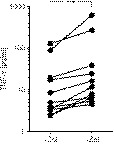
Zoledronate induces release of tumour necrosis factor (TNF)‐α by peripheral blood mononuclear cells (PBMCs). PBMCs from patients with acute sepsis (*n* = 11) were cultured in medium alone (– Zol) or treated overnight with zoledronate (+ Zol). Levels of TNF‐α released into the culture supernatants were determined by ELISA. Data were analysed using a Wilcoxon matched pairs signed rank test; ***P < 0·001.

Loss of HLA‐DR surface expression is a well‐known phenomenon in sepsis, as surrogate marker of immunosuppression and predictor of poor outcomes.^6^, ^7^, ^8^, ^9^ Here, monocytes in sepsis patients expressed lower levels of HLA‐DR and CD86 during the first 36 hr of presenting with acute illness, compared with healthy controls (Fig. 3), suggesting functional defects in those patients. In PBMC cultures, zoledronate treatment led to an upregulation of HLA‐DR as well as CD40 and CD64 on sepsis monocytes, which at least in the case of HLA‐DR and CD40 could be inhibited by the addition of neutralising reagents against IFN‐γ and TNF‐α (Fig. 4). In accordance with the role of TNF‐α in these stimulation experiments, treatment of PBMCs from sepsis patients with zoledronate led an increased release of TNF‐α into the culture supernatant (Fig. 5).

### Patients with positive blood cultures have lower responses to zoledronate

**Figure 6:**
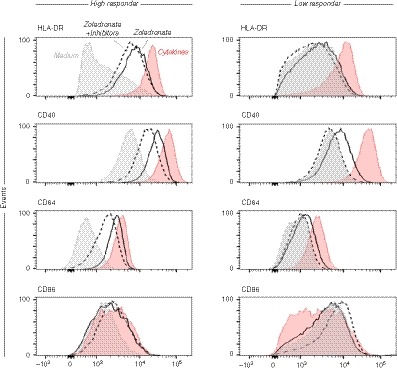
Patient to patient variation in the responsiveness of monocytes to zoledronate stimulation. Peripheral blood mononuclear cells (PBMCs) from patients with acute sepsis were cultured in medium alone (shaded histograms) or treated overnight with zoledronate in the absence (solid line) or presence of anti‐interferon (IFN)‐γ and sTNFR (dotted line; Inhibitors), or with a combination of recombinant IFN‐γ and tumour necrosis factor (TNF)‐α (red histograms; Cytokines). Data shown are representative of a ‘high responder’ (left panels) and a ‘low responder’ (right panel).

**Figure 7:**
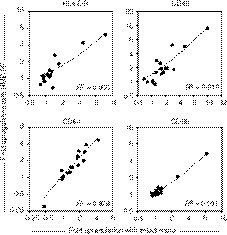
Responsiveness of sepsis monocytes to zoledronate and HMB‐PP. Peripheral blood mononuclear cells (PBMCs) from patients with acute sepsis (*n* = 16) were cultured in medium alone or treated overnight with either 10 µm zoledronate or 100 nm HMB‐PP. Monocyte activation was calculated as fold upregulation of the indicated surface markers (determined as mean fluorescence intensities), in relation to medium controls with a value of 1. Data were analysed using linear regression.

**Figure 8:**
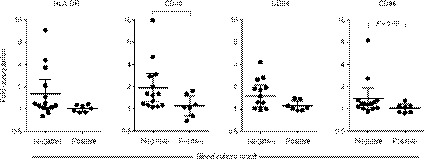
Responsiveness of sepsis monocytes to zoledronate depending on blood culture results. Peripheral blood mononuclear cells (PBMCs) from sepsis patients with microbiologically negative (*n* = 14) or positive (*n* = 7) blood cultures were incubated in medium alone or treated overnight with zoledronate. Monocyte activation was calculated as fold upregulation of the indicated surface markers, in relation to medium controls with a value of 1. Data were analysed using Mann−Whitney tests; asterisks indicate significant differences: *P < 0·05; **P < 0·01; ***P < 0·001.

Overall, there was substantial patient‐to‐patient variation with regard to the expression of surface markers on circulating monocytes and their potential to respond to zoledronate stimulation. While in some individuals zoledronate induced substantial upregulation of APC markers (‘high responders’), in others zoledronate treatment had only very little effect (‘low responders’; Fig. 6). This variable responsiveness did not correlate with the severity of the clinical symptoms as assessed by APACHE II scores (data not shown). In addition, it was independent of the actual γδ T‐cell stimulus provided *in vitro* as exposure to zoledronate and HMB‐PP induced similar responses (Fig. 7). However, monocytes from sepsis patients with systemic bacterial infections as confirmed by positive blood cultures showed only very little responsiveness to zoledronate, compared with sepsis patients with more localised infections marked by negative blood cultures (Fig. 8). Of note, there were no quantitative differences in peripheral Vγ9/Vδ2 T‐cell or monocyte levels between patients with negative and positive blood cultures that could have explained this phenomenon (data not shown).

## Discussion

The present study is the first demonstration that experimental exposure of human PBMCs to the anti‐bone resorption drug zoledronate drives upregulation of APC markers on monocytes from both healthy individuals and patients with acute sepsis. These findings extend our earlier investigations in γδ T‐cell–monocyte co‐cultures *in vitro* 13 and in zoledronate‐treated individuals with osteoporosis *in vivo*.^22^ Taken together, we conclude that zoledronate treatment has the potential, at least partially, to rescue aspects of the pronounced immunosuppression frequently observed in sepsis patients and may thereby help improve clinical outcomes.

Our data are consistent with the view that once taken up by monocytes, zoledronate causes intracellular accumulation of metabolites of the mevalonate pathway such as isopentenyl pyrophosphate, which is then sensed by Vγ9/Vδ2 T‐cells. Vγ9/Vδ2 T‐cells produce copious amounts of pro‐inflammatory cytokines like IFN‐γ and TNF‐α upon activation, which in turn affect the function of bystander cells in the microenvironment, including monocytes, neutrophils and local tissue cells.^16^, ^23^, ^24^ This model is supported by the present observation that neutralising the effects of IFN‐γ and TNF‐α in zoledronate‐treated PBMC cultures blocked the upregulation of HLA‐DR and CD40 on monocytes from both healthy individuals and patients with severe sepsis, and that exposure of sepsis PBMC to zoledronate led to an increased release of TNF‐α into the culture medium. While we were unable to confirm the cellular source of TNF‐α, our previous work suggests that TNF‐α is produced by both Vγ9/Vδ2 T‐cells and monocytes under such conditions.^13^, ^14^ Unfortunately, the limited amounts of patient blood available for the present study and the low numbers of Vγ9/Vδ2 T‐cells in most samples (with a median frequency of 0·58% among live PBMCs; data not shown) did not allow us to assess directly markers of successful Vγ9/Vδ2 T‐cell activation. These limitations notwithstanding, our findings support considerations to use aminobisphosphonates like zoledronate – or related γδ T‐cell‐stimulating treatments such as HMB‐PP analogues or anti‐BTN3 agonistic antibodies – as novel immunotherapies in sepsis patients. However, care should be taken to balance such potential benefits with the risk of possible side‐effects. While the pro‐inflammatory nature of γδ T‐cell responses will be able to boost APCs and thus help rescue the underlying immunosuppression, the very nature of such responses may simultaneously give rise to an acute phase‐like reaction, which will have to be carefully mitigated.^22^, ^25^, ^26^ Also, while zoledronate treatment clearly has stimulatory effects on monocytes, others have shown that zoledronate‐treated monocytes and macrophages may become targets for γδ T‐cell‐mediated killing,^27^, ^28^ thereby potentially limiting the therapeutic window.

Strikingly, a proportion of sepsis patients studied here did in fact not show a demonstrable response to zoledronate, predominantly patients with microbiologically confirmed bloodstream infections. There was generally good correlation between responses to zoledronate and the microbial metabolite HMB‐PP serving as control, thereby ruling out a potential defect in low responders affecting zoledronate uptake by monocytes and/or its intracellular mode of action. Low responsiveness to zoledronate in certain individuals has been described before, most notably in patients with chronic lymphocytic leukaemia or multiple myeloma.^29^, ^30^ The underlying mechanism for the unresponsiveness in blood culture‐positive sepsis patients remains unclear but may involve a systemic dysfunction of γδ T‐cells and/or monocytes as a result of the severe infection, differences in the activity of the mevalonate pathway in monocytes, elevated levels of regulatory T‐cells or immunosuppressive molecules, and genetic and environmental factors. More research is needed to be able to stratify patients with regard to the underlying pathology, nature of the infective organism (e.g. with regard to their potential to produce HMB‐PP), treatment options and general demographics, and identify those sepsis patients who may benefit most from a zoledronate‐based therapy.

## Disclosures

The authors have no conflicts of interest.

## Author contributions

MPM and ME designed the study, LR, RJB and SEB performed the experiments, LR, RJB and ME discussed and analysed the data, and ME wrote the paper. All authors contributed to and approved the final version.
